# Plasmid DNA Vaccine Co-Immunisation Modulates Cellular and Humoral Immune Responses Induced by Intranasal Inoculation in Mice

**DOI:** 10.1371/journal.pone.0141557

**Published:** 2015-11-06

**Authors:** Deborah F. L. King, Paul F. McKay, Jamie F. S. Mann, C. Bryn Jones, Robin J. Shattock

**Affiliations:** Mucosal Infection and Immunity Group, Department of Infectious Diseases, Division of Medicine, Imperial College, London, United Kingdom; University of Massachusetts Medical School, UNITED STATES

## Abstract

**Background:**

An effective HIV vaccine will likely require induction of both mucosal and systemic cellular and humoral immune responses. We investigated whether intramuscular (IM) delivery of electroporated plasmid DNA vaccine and simultaneous protein vaccinations by intranasal (IN) and IM routes could be combined to induce mucosal and systemic cellular and humoral immune responses to a model HIV-1 CN54 gp140 antigen in mice.

**Results:**

Co-immunisation of DNA with intranasal protein successfully elicited both serum and vaginal IgG and IgA responses, whereas DNA and IM protein co-delivery did not induce systemic or mucosal IgA responses. Cellular IFNγ responses were preserved in co-immunisation protocols compared to protein-only vaccination groups. The addition of DNA to IN protein vaccination reduced the strong Th2 bias observed with IN protein vaccination alone. Luminex analysis also revealed that co-immunisation with DNA and IN protein induced expression of cytokines that promote B-cell function, generation of T_FH_ cells and CCR5 ligands that can reduce HIV infectivity.

**Significance:**

These data suggest that while IN inoculation alone elicits both cellular and humoral responses, co-administration with homologous DNA vaccination can tailor these towards a more balanced Th1/Th2 phenotype modulating the cellular cytokine profile while eliciting high-levels of antigen-specific antibody. This work provides insights on how to generate differential immune responses within the same vaccination visit, and supports co-immunisation with DNA and protein by a mucosal route as a potential delivery strategy for HIV vaccines.

## Introduction

Development of an effective vaccine to prevent HIV-1 infection is critical to control the spread of HIV. Although the mechanistic correlates of HIV protection remain to be defined, recent studies have started to reveal key cellular and humoral responses likely required for an effective vaccine. Data from the rhesus macaque model indicates that HIV-1 Env specific neutralizing antibodies protect against mucosal [1–3] and intravenous SHIV challenge [4, 5], as well as from heterologous SIV challenge [6] reducing the pathogenic effects of infection such as CD4+ T-cell loss, and the viral set-point [1, 4, 6]. Data from the RV144 clinical vaccine trial demonstrated that a reduced risk of HIV-1 infection correlated with binding titer of IgG_3_ antibodies to HIV Env variable regions 1 and 2 (V1/V2), reinforcing the role of Env-specific antibodies in preventing infection [7]. Nevertheless cellular immune responses, specifically the magnitude and breadth of Gag-specific responses, are also thought to be critical in controlling virus replication and preventing loss of CD4+ T cells [6, 8]. Thus current data suggest that an effective vaccine will likely need to induce both cellular and humoral immune responses to reduce the rate of HIV-1 infection and control virus replication in the advent of breakthrough infection.

Identifying an appropriate strategy to induce such cellular and humoral immune responses both systemically and at mucosal portals of entry remains critical in developing an HIV vaccine. Previous studies by our group using BALB/c mice have shown that IN immunization with a model HIV-1 antigen, gp140, adjuvanted by TLR4 agonist Glucopyranosyl Lipid A (GLA/MPLA) was optimal for the induction of humoral antigen-specific responses in the vagina [9, 10]. Furthermore, we and others have also demonstrated DNA vaccination to be effective at inducing both cellular and humoral immune responses to HIV/SIV DNAs in mice [11, 12] and macaques, and can reduce virus load upon challenge [13–15]. Subsequent protein or viral-vector boosting can improve the immunogenicity of DNA vaccination [16] and has been shown to provide protection from homologous SHIV challenge in macaques [17]. Thus the combination of DNA vaccination and protein boosting is a promising strategy for an effective HIV vaccine. However, sequential vaccination by multiple routes in a standard prime-boost regimen is likely to result in a protracted vaccination schedule that may be difficult to implement in resource poor settings. Recent studies have suggested that co-immunization of DNA and protein antigens by IM routes can improve humoral immune responses in mice, rabbits and macaques [18–20]. In this context we chose to investigate the impact of multiple dosing strategies given simultaneously given in a conventional prime-boost-boost schedule, with a view to regimen shortening associated with sequential use of multiple products. The aim of this study was to determine whether a plasmid DNA vaccine and protein vaccinations administered by IN and IM routes could be co-administered to induce systemic and mucosal humoral immune responses to a model HIV-1 CN54 gp140 antigen.

## Materials and Methods

### Antigens and Adjuvants

HIV-1 Env protein for vaccination consisted of recombinant HIV-1 CN54 gp140 envelope protein from the clade C/B' strain 97/CN/54, the envelope protein is of clade C origin (Polymun Scientific, Austria) [21]. The gp140 protein is derived from gp160, rendered soluble by truncation of the transmembrane domain and cytoplasmic tail of gp41, but retaining HR1 and HR2 domains. DNA vaccination utilized a plasmid encoding a codon optimized sequence of CN54 gp140 (GeneArt, Invitrogen, UK) and a CMV enhancer/promoter with a human T-cell leukemia type 1 regulatory element to drive transgene expression, obtained from the UK-HIV Vaccine Consortium (UK-HVC). Protein vaccinations were adjuvanted with Monophosphoyl Lipid A (MPLA, Sigma-Aldrich) a TLR4 ligand. MPLA was reconstituted in a PBS / 20% Dimethyl Sulfoxide (DMSO) solution to a final MPLA concentration of 4 mg/ml.

### Animals

Female BALB/c mice were provided by Harlan (UK). Seven groups of mice aged 6–8 weeks old, were placed into groups of n = 8 and housed in a fully acclimatized room. All animals were handled, procedures performed and the study carried out in strict accordance with the conditions of a project licence granted under the UK Home Office Animals (Scientific Procedures) Act 1986. The protocol was peer reviewed and approved by the Imperial College Ethical Review Process (ERP) Committee and any amendments were peer-reviewed and approved by the Imperial College Animal Welfare and Ethical Review Body (AWERB). Animals received minimal handling, procedures were performed under isoflourane anesthesia when appropriate, and all efforts were made to minimize suffering. Animal condition and health was monitored daily. Animals were culled using a schedule 1 method and death confirmed before autopsy. Food and water were supplied *ad libitum*.

DNA vaccinations were given IM into the quadriceps using 20 μg HIV-1 CN54 gp140-encoding DNA in a total volume of 30 μl in sterile water. Mice were then electroporated (EP) using 5 mm spaced electrodes. Electric pulses were delivered using the ECM 830 Square Wave Electroporation System (BTX). Three pulses of 100 V each, followed by three pulses of the opposite polarity, were delivered to each injection site with each pulse (P_ON_) lasting 50 ms and an interpulse (P_OFF_) interval of 50 ms. Protein vaccinations were delivered either by IM injection into the quadriceps or IN. For both IM and IN vaccinations 10 μg recombinant HIV-1 CN54 gp140 protein (in PBS) was combined with 20 μg (5 μl) MPLA (in a PBS / 20% DMSO solution) and made up to a total volume of 25 μl with sterile PBS. Therefore the final vaccine solution contains 4% DMSO. Animals received either three simultaneous vaccinations (group 1 DNA + IN + IM Protein), two vaccinations (groups 2 (DNA + IN), 3 (DNA + IM Protein), 4 (IN + IM Protein)), or one vaccination (groups 5 (DNA), 6 (IN), 7 (IM Protein)). Animals were vaccinated at three time points and euthanized one week after the last vaccination (Table 1).

**Table 1:** Vaccination schedule.

Group	Prime (week 0)	Boost 1 (week 3)	Boost 2 (week 7)
1 (n = 8)	DNA + IN + IM	DNA + IN + IM	DNA + IN + IM
2 (n = 8)	DNA + IN	DNA + IN	DNA + IN
3 (n = 8)	DNA + IM	DNA + IM	DNA + IM
4 (n = 8)	IN + IM	IN + IM	IN + IM
5 (n = 8)	DNA	DNA	DNA
6 (n = 8)	IN	IN	IN
7 (n = 8)	IM	IM	IM

Animals were vaccinated at three time points with the indicated vaccine combinations. DNA = intramuscular injection with HIV-1 gp140-encoding plasmid with electroporation, IM = Intramuscular injection with HIV-1 CN54 gp140 Env protein with MPLA, IN = Intranasal delivery of HIV-1 CN54 gp140 Env protein with MPLA.

### Sampling and processing

Animals were sampled pre-vaccination (week 0) and one week after each vaccination (weeks 1, 4 and 8). Tail bleeds were collected from mice without anti-coagulant and centrifuged in a Heraeus Biofuge pico (Fisher, UK) at 20,000 g for 10 min at room temperature. The serum was harvested and transferred into fresh 0.5 ml micro-centrifuge tubes, and stored at -20°C. Murine vaginal lavage samples were taken using three 25 μl washes/mouse with PBS. Lavage samples were incubated for 30 min with 4μl protease inhibitor (Roche Diagnostics, Germany) before centrifuging at 14,000 rpm for 10 min at 4°C. The fluid supernatant from these treated samples were then transferred into fresh 0.5ml micro-centrifuge tubes, and stored at -20°C

To assess IFN-γ and IL-2 T-cell responses and antigen-specific IgG and IgA B cell responses, lymphocyte cultures were made from spleens of immunized mice. Briefly, mice were euthanized one week after the third vaccination, and their spleens removed aseptically and placed into individual 15 ml tubes (Greiner, UK) containing 5 ml RPMI 1640 medium (Sigma Aldrich Ltd, UK). Single cell suspensions were made from the spleens by grinding them through a 70 μm Nylon Cell Strainers (BD Falcon, UK) using a sterile syringe plunger to yield a single cell suspension. The cell suspensions were then centrifuged at 350 g for 10 min. The supernatants were decanted and the pelleted cells re-suspended in 5ml ACK lysis buffer (Gibco, UK) for 5 min at room temperature. The cell suspensions were vortexed and centrifuged at 350 g for 10 min. The pelleted cells were decanted and re-suspended in 5 ml RPMI 1640 medium. This step was repeated three times with the cells resuspended in complete RPMI and then filtered through a 100 μm Filcon unit (BD Biosciences, UK). The cells were counted using a hemocytometer and resuspended at 5x10^6^/ml in complete RPMI 1640.

### ELISA

Serum and vaginal lavage samples were tested for antigen-specific IgG, IgG1, IgG2a and IgA by semi-quantitative ELISA [12]. ELISA plates were coated with 100 μl/well of HIV Env gp140 antigen, at a concentration of 5 μg/ml diluted in sterile PBS. Plates were then sealed and incubated overnight at 4°C. The plates were then washed four times by PBST (Invitrogen, UK) and blocked by adding 200 μl per well of assay buffer (PBST + 1% BSA). The plates were then sealed and incubated for 1 hour at 37°C. After the incubation, the plates are washed 4 times with 300 μl of PBST. Serum samples were tested at dilutions of 1:100, 1:1000 and 1:10000 in triplicate, vaginal lavage samples were tested for specific antibody at dilutions of 1:50, 1:250 and 1:1250 in triplicate, 50 μl sample per well was used. Vaginal lavage samples were tested for total IgG and IgA at 1:100, 1:1000 and 1:10000. Plates were than resealed and incubated for 1 hour at 37°C. The plates were washed 4 times with 300 μl of PBST per well, and 100 μl/well of detection antibodies anti-mouse IgG-HRP, anti-mouse IgG1-HRP, anti-mouse IgG2a-HRP or IgA-HRP (Southern Biotech) were added at 1:4000 dilution in sample diluent. Plates were incubated for 1 hour at 37°C before 4 washes with 300 μl PBST. Plates were developed using the addition of 50 μl/well TMB and the reaction stopped after 5 min using 50 μl/well Stop solution (Insight Biotechnologies, UK). Standards on the plate consisted of coating with 100μl anti-mouse Kappa (1:3200) and Lambda (1:3200) light chain (Serotec, UK), blocking as before and then adding 50 μl of IgG (Southern Biotech, UK) or IgA (Southern Biotech, UK) in a 5-fold dilution series with a starting concentration of 1000 ng/ml. Detection and development of standard-containing wells was the same as the sample wells, as described above. The absorbance was read on a Microplate reader (VersaMax—Molecular Devices) using SoftMax Pro GxP v5 software.

### Avidity assay

The avidity indices of serum samples were determined by their antibody-antigen binding resistance to 8 M urea [11]. Briefly, ELISA plates were prepared and blocked as described above. Serum samples were diluted to give an OD450 nm readout between 1.0 and 1.5 in endpoint ELISA and were added to two sets of triplicate wells to incubate for 1 h at 37°C. The wells were then washed three times with either PBST or 8 M urea in PBST, before incubating with an anti-mouse IgG-HRP secondary antibody. The plates were washed and developed with TMB as described above. The absorbance was read on a Microplate reader (VersaMax—Molecular Devices) using SoftMax Pro GxP v5 software. The avidity index was calculated as the percentage of average urea treated OD450 nm/average PBS-Tween OD450 nm. Antisera with index values exceeding 50% were ascribed to have high avidity, 30–50% were ascribed as intermediate avidity and <30% were low avidity binding.

### IgG and IgA ELISpot

Antigen-specific IgG and IgA ELIspot (Mabtech AB) responses were detected in splenocytes, according to manufacturer’s instructions. Briefly, plates were coated with either PBS alone (negative control) or with 5 μg/ml HIV-1 CN54 gp140 and incubated overnight at 4°C. Plates were then washed five times with sterile PBS and blocked for 30 minutes at room temperature with 200 μl/well of complete RPMI. After blocking, medium was removed and 2.5x10^5^ cells were added to each well. For measurement of total IgG and IgA, wells were coated with anti-IgG or anti-IgA coating antibody at 15 μg/ml and 10 μg/ml respectively, and cells were added at a concentration of 7.5x10^4^/well. All conditions were performed in triplicate wells. The plates were incubated for 38 hours at 37°C supplemented with 5% CO2. To detect spots, 100 μl of 1 μg/ml biotinylated anti-IgA or biotinylated anti-IgG were added to wells for 2 hours before washing and incubating with Streptavidin-HRP for 1 hour. The plates were washed as before and 100 μl/well of TMB substrate added. ELIspot plates were read on an AID iSpot plate reader using AID version 6 software.

### IFN γ and IL-2 ELISpot

Antigen-specific IFNγ and IL-2 ELISpot assays (Mabtech AB) were performed on splenocytes in response to an HIV-1 CN54 gp140 peptide pool, according to the manufacturer’s instructions. Briefly, IL-2 plates were coated with IL-2 detection antibody at 15 μg/ml, 100 μl/well overnight at 4°C. IFNγ plates were pre-coated. The following day plates were washed five times in sterile PBS and blocked for 30 min using complete RPMI. The media was removed from the plate and the 2x10^5^ cells were added to each well in a volume of 50 μl. Next, gp140 peptide pools were added at a final concentration of 2.5 μg/ml to stimulate the cells to assess antigen-specific responses, peptides were divided into two pools, pool 1 covering amino acids 1–78, and pool 2 covering amino acids 79–156 of HIV-1 CN54 gp140, and consisted of 15-mers, overlapping by 11 aa. As controls, un-stimulated (media only) and PHA-stimulated (5 μg/ml) wells were included. All conditions were set up in triplicate wells. The plates were incubated for 18 hours at 37°C supplemented with 5% CO2. To detect spots, biotinylated anti-IFN-γ or anti-IL-2 antibody was added at 1 μg/ml for 2 hours before washing and incubating with streptavidin-HRP for 1 hour. The plates were washed as before and 100 μl/well of TMB substrate added. ELIspot plates were read on an AID iSpot plate reader using AID version 6 software.

### Cytokine detection in multiplex bead assay

Antigen-specific cytokine responses were measured in splenocytes using Bio-Plex Pro mouse cytokine standard 23-plex, group 1 (Bio-Rad). Freshly isolated splenocytes were stimulated with 5μg/ml recombinant gp140 protein, supernatant was harvested after 3 days of culture and stored at -20°C for analysis of cytokine production. Culture supernatants were simultaneously assessed for the production of the following cytokines: IL-1α, IL-1β, IL-2, IL-3, IL-4, IL-5, IL-6, IL-9, IL-10, IL-12p40, IL-12p70, IL-13, IL-17A, Eotaxin, G-CSF, GM-CSF, IFNγ, MCP-1, MIP-1α, MIP-1β RANTES and TNFα according to manufacturer’s instructions. Plates were read using the Luminex 100 system (Luminex) and data were analysed using the Bioplex Manager version 4.0 software (Bio-Rad). Samples that exceeded the upper limit of detection were allocated the highest standard value to allow data to be plotted.

### Statistical Analysis

Comparisons of responses between all groups were made using One-way ANOVA with Tukey’s post test using Prism v4 software for all assays other than Luminex cytokine array. For Luminex cytokine responses, Kruskal-Wallis with Dunn’s multiple comparison was used to analyse data.

## Results

### IN protein vaccination alone or combined with DNA enhances levels of HIV-specific IgG and IgA in the serum of vaccinated animals

**Fig 1:**
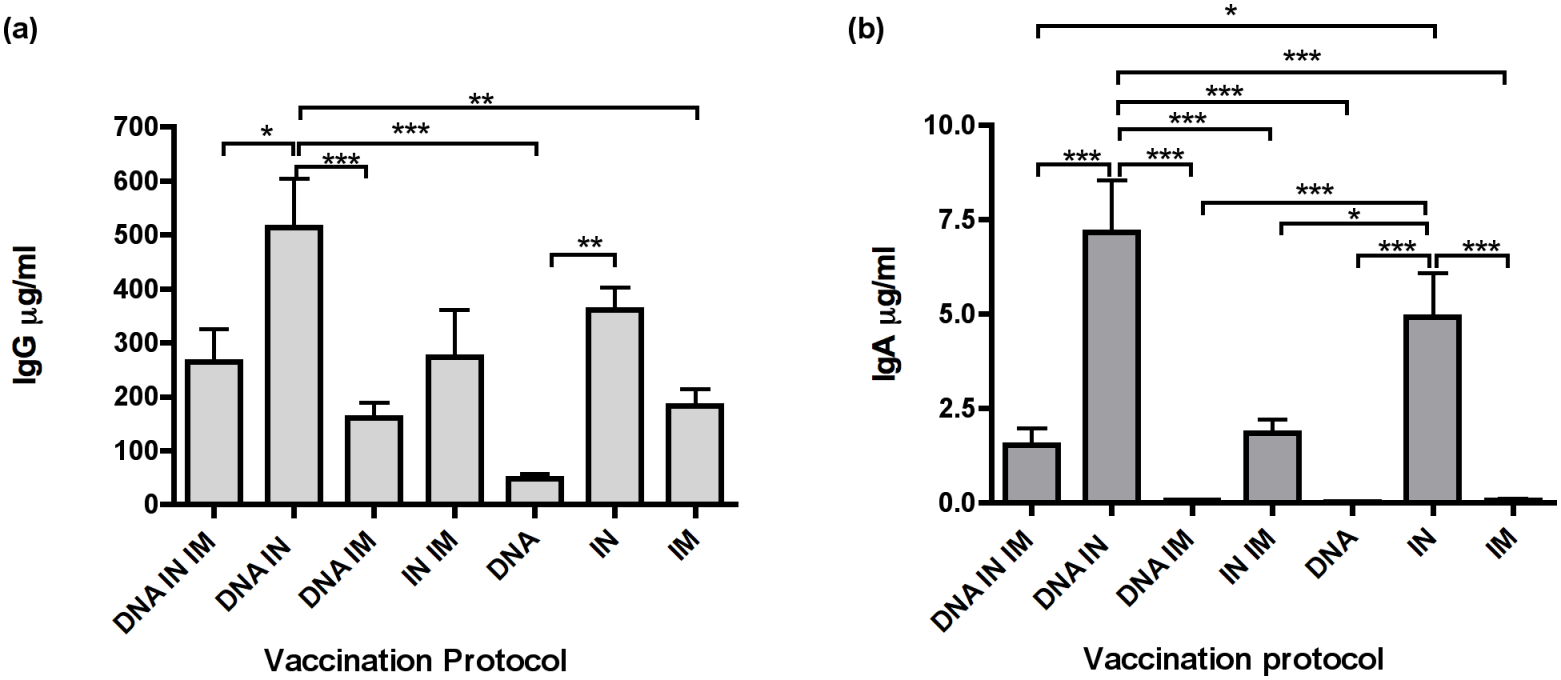
IN and DNA + IN regimens induce the highest serum IgG and IgA responses after three vaccinations. Mice were vaccinated three times with the indicated vaccine modalities, serum samples taken 1 week after the last vaccination (week 8) were tested for antigen-specific IgG (a) and IgA (b) by ELISA. Data were analysed using one-way ANOVA with Tukey’s post test, * P <0.05, ** P < 0.01, *** P < 0.001.

Initial studies were performed to assess the impact of gp140 encoding plasmid DNA and recombinant gp140 protein vaccinations adjuvanted with MPLA delivered by the IN and IM routes, either alone or as co-immunisations (Table 1). Serum antibody responses were compared one week after each vaccination. When the kinetics of response were observed over time, IM protein alone and IN + IM protein groups induced the highest serum antigen-specific IgG responses after the first vaccination (S1a Fig), however this early lead was not maintained and analysis one week after the final vaccination demonstrated that IN alone and DNA + IN groups had the highest levels of antigen-specific serum antibody (Fig 1a).

After three vaccinations HIV-1 gp140-specific IgG was observed in all groups of animals receiving vaccination by a single modality and route (DNA only, IN protein only, IM protein only), with significantly higher antibody levels observed after IN vaccination (361 μg/ml ± 40.8), compared to DNA vaccination (48 μg/ml ± 8.1). When two routes of vaccination were combined the highest antibody levels were observed in group 2, receiving DNA + IN protein vaccinations (513 μg/ml ± 90.9), and this was significantly higher than those observed when DNA was combined with IM protein delivery (160 μg/ml ± 28.5), or with IM protein alone (182 μg/ml ± 32.5). Combination of IN and IM protein routes did not significantly change the amount of antigen-specific IgG compared to IN delivery alone. Comparison of three simultaneous vaccinations (DNA + IN + IM protein) with DNA + IN demonstrated that inclusion of IM protein vaccination significantly reduced antigen-specific IgG in serum (Fig 1a).

IgA was not detected in any of the vaccinated groups until after the second vaccination (week 4, S1c Fig), and was observed at very low levels in protocols lacking IN antigen delivery (Fig 1b). The highest antigen-specific IgA levels were recorded in IN only and DNA + IN vaccination protocols (4.9 μg/ml ± 1.2, and 7.2 μg/ml ± 1.4 respectively). IN vaccination induced significantly higher levels of IgA compared to DNA or IM protein only, when IN was combined with IM protein, or when DNA + IM protein routes were used. DNA + IN induced significantly higher levels of antigen-specific IgA compared to all other protocols except IN alone (Fig 1b). Similar to the IgG response, addition of an IM protein vaccination to the DNA + IN vaccinations resulted in a reduction in serum IgA compared to DNA + IN vaccination (1.5 μg/ml ± 0.4 vs 7.2μg/ml ± 1.4) (Fig 1b).

Thus, IN alone or DNA + IN vaccination were the most effective routes and modalities for inducing serum IgG and IgA responses.

### IN protein alone, or DNA + IN vaccination induce vaginal antigen-specific IgA responses

**Fig 2:**
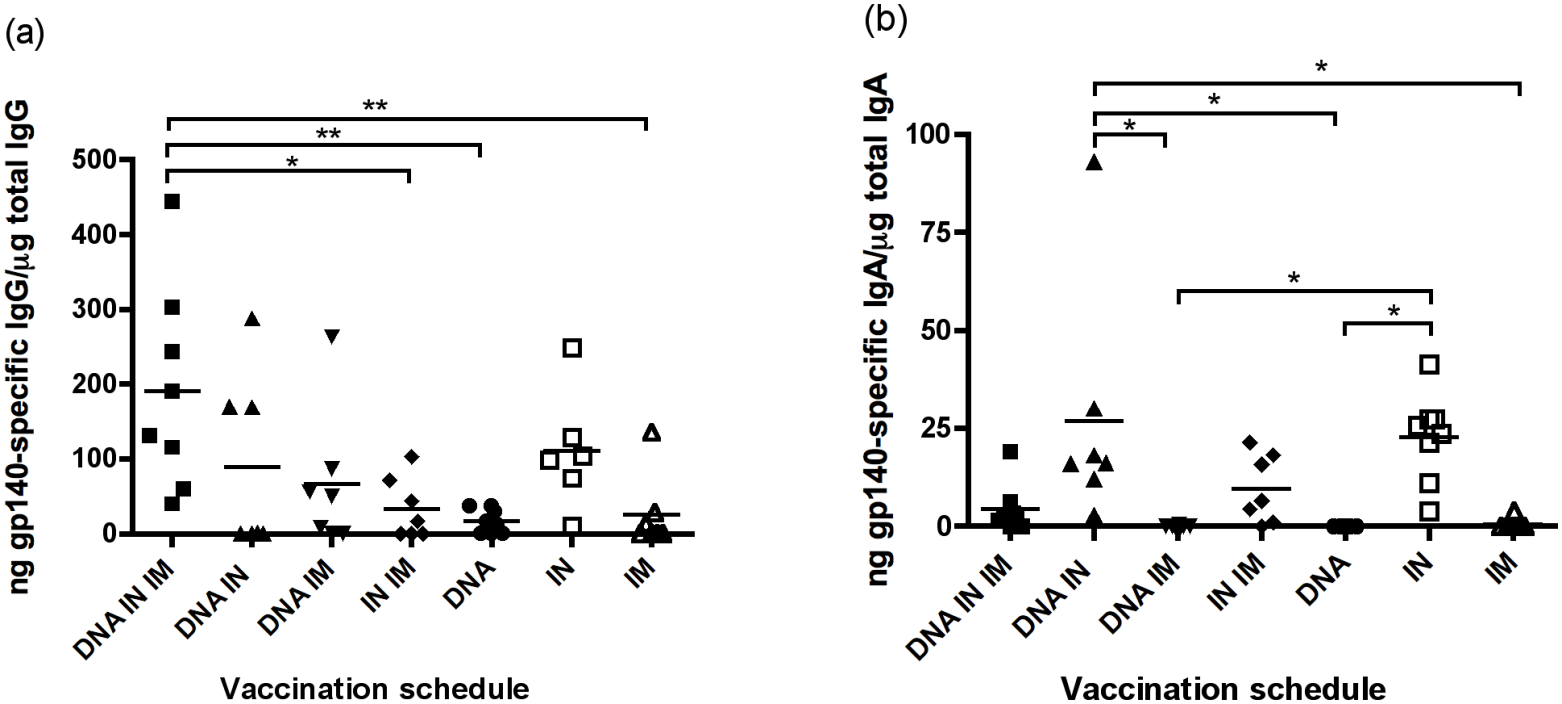
Detection of mucosal antigen-specific IgG and IgA responses in the vagina. Mice were vaccinated three times and vaginal lavage samples were taken 1 week after the last vaccination and tested for antigen-specific IgG (a) and IgA (b) by ELISA. Results are expressed as specific antibody (ng)/total antibody (μg). One-way ANOVA with Tukey’s post test was used to analyse data. * P <0.05, ** P < 0.01, *** P < 0.001.

The presence of antigen-specific antibody at mucosal portals of entry, are thought to be necessary to prevent HIV infection. Therefore we assessed the ability of the various vaccination regimens to elicit anti-HIV humoral responses in mucosal secretions. Antigen-specific and total IgG and IgA were measured in vaginal lavage samples by ELISA one week after the last vaccination. Similar levels of antigen-specific IgG were observed in groups 1, 2, 3, and 6 (Table 1) with the highest levels observed in group 1 (DNA + IN + IM protein, 191 ng/μg total Ab ± 47.95). Antigen-specific IgG in this group was significantly higher than in the IN + IM protein group (33.56 ng/μg total Ab ± 15.44), DNA alone (16.76 ng/μg total Ab ± 5.75) and IM protein alone (25.45 ng/μg total Ab ± 18.99) groups (Fig 2a).

In groups receiving a single route of immunisation, vaginal antigen-specific IgA was highest in the IN group (22.7 ng/μg total Ab ± 3.30). When two simultaneous vaccinations were given, DNA + IN vaccination resulted in the highest levels of antigen-specific IgA (26.91 ng/μg total Ab ± 11.43) compared to other co-immunisation groups. Both IN alone and DNA + IN groups had significantly more IgA in vaginal samples than DNA alone (where vaginal antigen-specific IgA was not detectable) or DNA + IM protein groups (Fig 2b).

### All vaccination protocols except DNA alone induce Th2 biased responses

**Fig 3:**
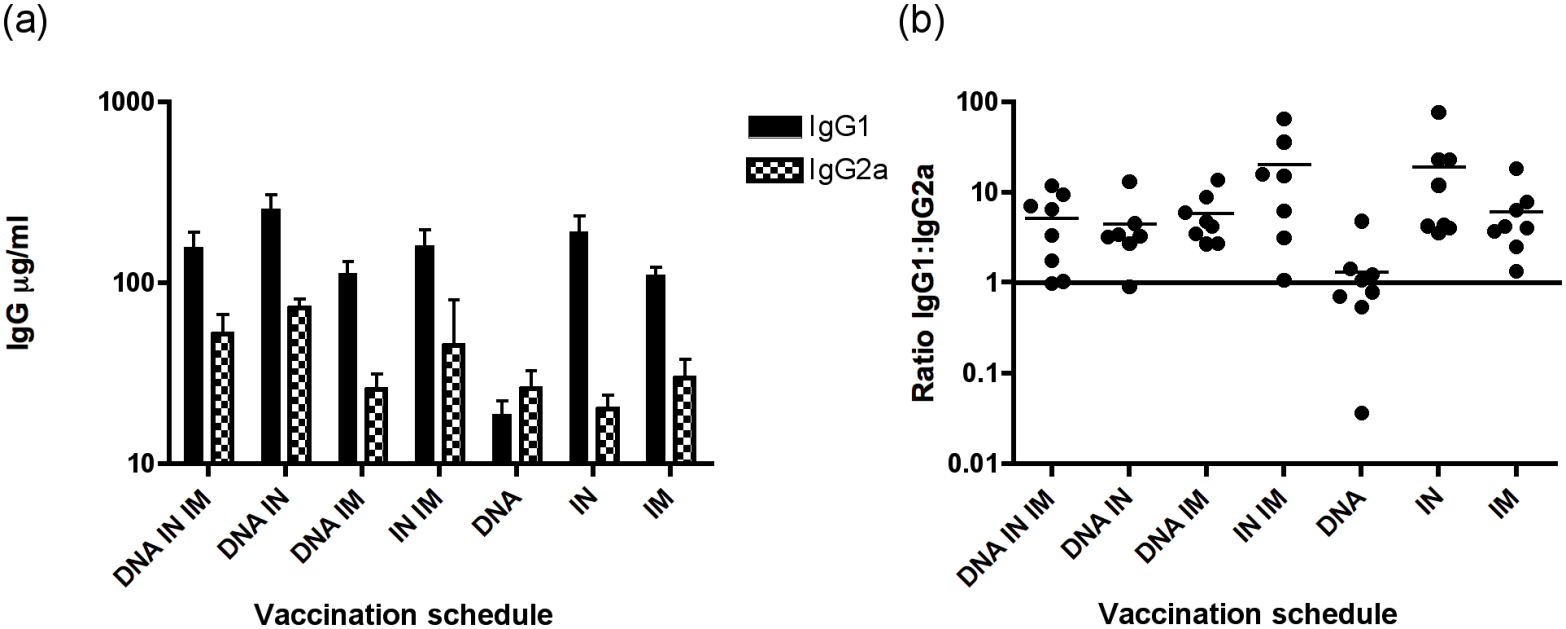
DNA vaccination induces Th1 skewed responses. Levels of IgG1 and IgG2a were assessed in serum one week after the last vaccination (week 8) by ELISA (a) and IgG1:IgG2a ratios were calculated as a surrogate of Th1/Th2 bias (b).

Analysis of IgG subclasses was performed on serum samples by ELISA measurement of IgG1 and IgG2a. Specific IgG1:IgG2a ratios were calculated as a surrogate of Th1/Th2 biasing of humoral responses. In all groups other than DNA alone, IgG1 levels were higher than IgG2a, suggesting Th2 biased responses (Fig 3a; S2a and S2c Fig). The highest ratios were observed in IN and IN + IM protein groups, giving highly skewed Th2 responses. In the DNA alone group, at weeks 1 and 4, higher levels of IgG2a than IgG1 were recorded, with ratios of IgG1:IgG2a of 0.64 and 0.54 respectively, reflecting a Th1 biased response (S2a–S2d Fig). At week 8 this group exhibited similar levels of IgG1 and IgG2a, with a ratio of IgG1:IgG2a of 1.32 (Fig 3b). Combination of DNA with IN vaccination resulted in increased amounts of IgG2a, with similar amounts of IgG1, which reduced the IgG1:IgG2a ratio from 18.9 in IN alone to 4.4 in the DNA + IN group, modestly reducing the strong Th2 bias observed with IN vaccination alone. Serum and vaginal antibody responses are summarised in Table 2.

**Table 2:** Mean humoral responses for each vaccination protocol.

Group	Serum (μg/ml)		Vaginal		IgG1:IgG2a	Avidity
			(ng spec/ μg total)			
	IgG	IgA	IgG	IgA		
DNA IN IM	264.5	1.5	**191.0**	4.4	5.2	23.6
DNA IN	**513.0**	**7.2**	89.7	**26.9**	4.4	18.5
DNA IM	160.2	0.1	66.3	0.1	5.8	29.6
IN IM	272.7	1.8	33.6	9.6	20.2	30.2
DNA	48.3	0.0	16.8	0.0	1.3	29.4
IN	361.3	4.9	111.1	22.7	18.9	35.5
IM	182.1	0.1	25.5	0.5	6.0	**39.1**

### Combination of DNA and IN protein vaccination induces the highest frequency of antigen-specific IgA secreting B cells

**Fig 4:**
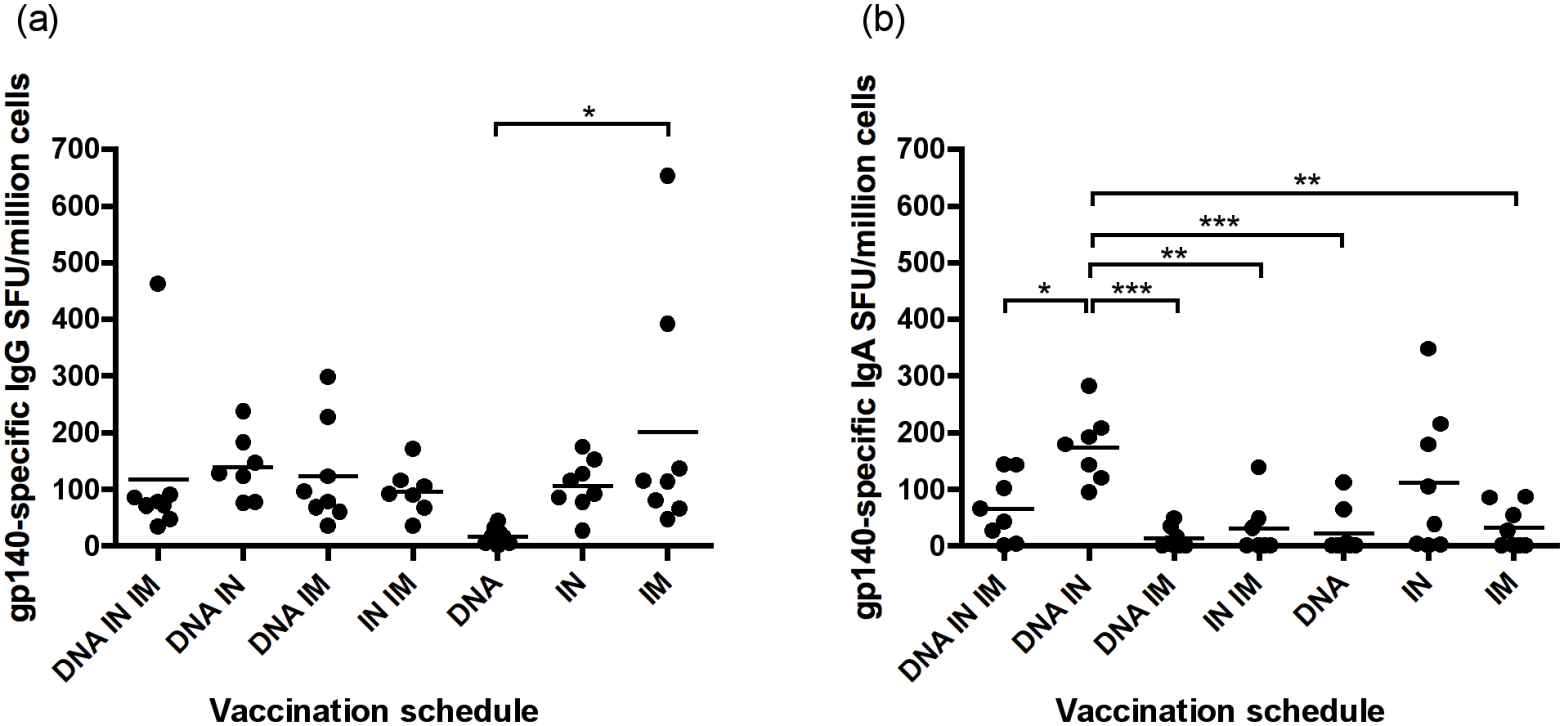
The highest frequency of IgA-secreting cells was observed after simultaneous DNA and IN vaccination. The frequency of antibody secreting IgG (a) and IgA (b) splenocytes were assessed using commercial ELIspot kits. One-way ANOVA with Tukey’s post test was used to analyse data * P <0.05, ** P < 0.01, *** P < 0.001.

Evaluation of splenocyte cultures by antigen-specific ELISpot demonstrated that all protocols induced antigen-specific IgG+ B cells, with similar frequencies of spot-forming units (SFU) observed in all protocols. In agreement with serum antibody data, the vaccination protocol with DNA alone induced the lowest frequency of IgG+ B cells (17.5 SFU ± 5.24), and this was significantly less than those observed with IM protein vaccination only, which induced the highest frequency of IgG+ cells (200.2 SFU ± 75.27) (Fig 4a). There was a positive correlation between gp140-specific IgG in the serum at week 8 and frequency of IgG+ B cells observed in ELIspot analysis (r^2^ = 0.1487, P = 0.004, S3a Fig).

Determination of IgA+ B cells in splenocyte cultures showed the highest frequency of cells in the DNA + IN and IN alone groups, with the DNA + IN group having significantly higher numbers of IgA+ B cells than all other groups except IN only. This is in agreement with levels of antigen-specific IgA observed in the serum. The addition of an IM protein vaccination to the DNA + IN protocol significantly reduced the frequency of IgA+ cells recorded, which was again in agreement with the observed reduction in serum IgA and there was a positive correlation between serum gp140-specific IgA at week 8 and frequency of IgA+ B cells observed in ELIspot analysis (r^2^ = 0.4432, P < 0.0001, S3b Fig) (Fig 4b).

### DNA vaccination induces potent IFNγ responses in splenocytes

**Fig 5:**
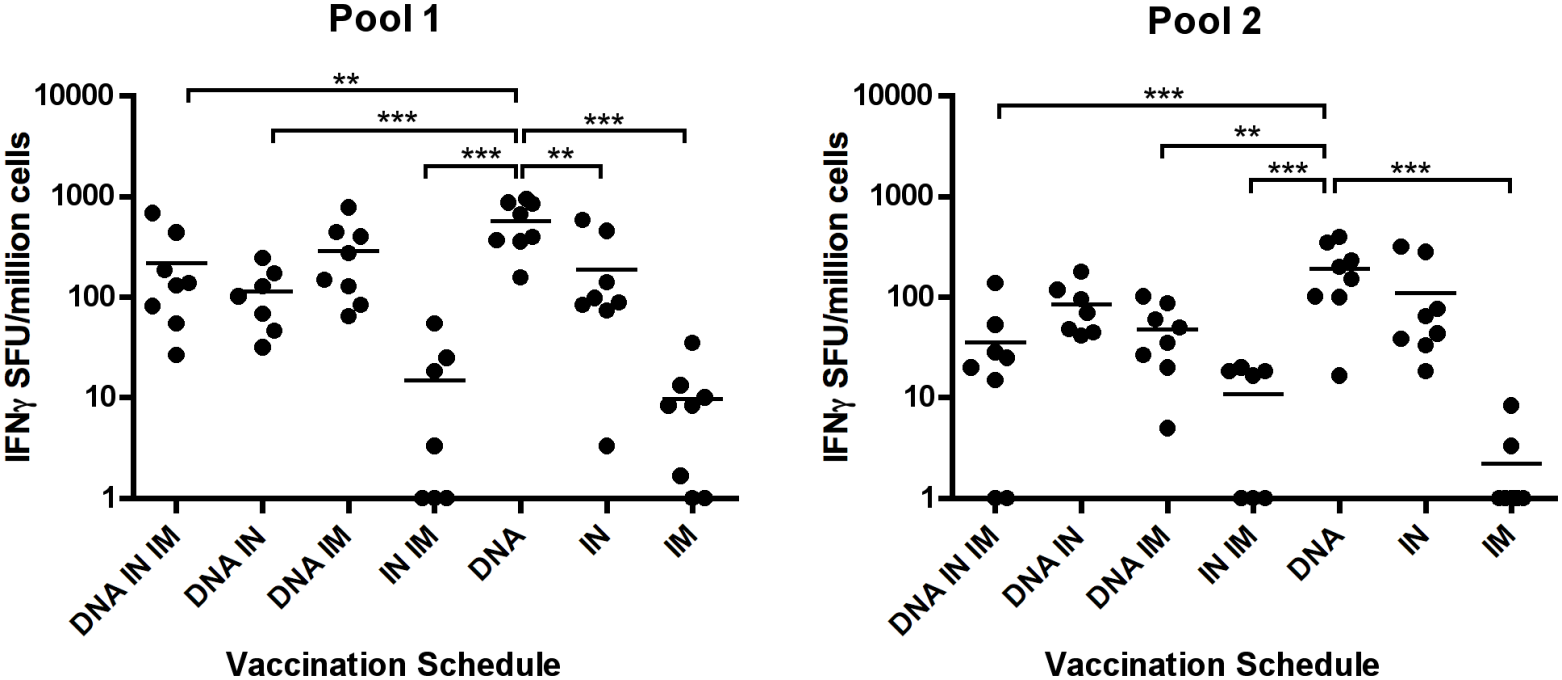
DNA vaccination induces potent IFNγ responses to HIV-1 Env peptides. Animals were vaccinated three times by the routes indicated. One week after the final vaccination splenocytes were cultured with HIV Env-specific peptide pools, and IFNγ-producing cells were measured using commercial ELIspot kits. Negative responses were assigned a value of 1 to allowing plotting on a log scale. One-way ANOVA with Tukey’s post test was used to analyse data * P <0.05, ** P < 0.01, *** P < 0.001.

To evaluate the ability of various vaccination regimens to elicit antigen-specific T cell responses we evaluated splenocyte cultures by IFNγ ELISpot. Splenocytes from animals euthanized one week after the third vaccination were stimulated overnight with two peptide pools covering the whole HIV-1 CN54 Env sequence, peptides were 15mers overlapping by 11: pool 1 corresponds to amino acids 1–78 (covering C1, V1/V2, C2, V3 and part of C3) and pool 2 corresponds to amino acids 79–156 (covering part of C3, V4, C5, V5 and C5 regions) of Env gp140. Responses to pool 1 were higher in all groups than responses to pool 2. The highest frequency of IFNγ+ cells was observed in DNA only immunised animals (575 SFU ±103.4 for pool 1, 193.1 SFU ± 45.5 for pool 2). The lowest frequency of IFNγ+ cells was observed in the IM only immunisation group with 9.583 SFU ± 4.031 (pool 1) and 1.458 SFU ± 1.065 (pool 2). The responses in the DNA only group were significantly higher than all other groups except DNA + IM protein for pool 1. For pool 2, the DNA only group had a similar frequency of IFNγ+ cells as the IN only and DNA + IN groups, and significantly more responding cells than all other groups (Fig 5).

### IN immunisation results in high levels of IL-2 responding T-cells

**Fig 6:**
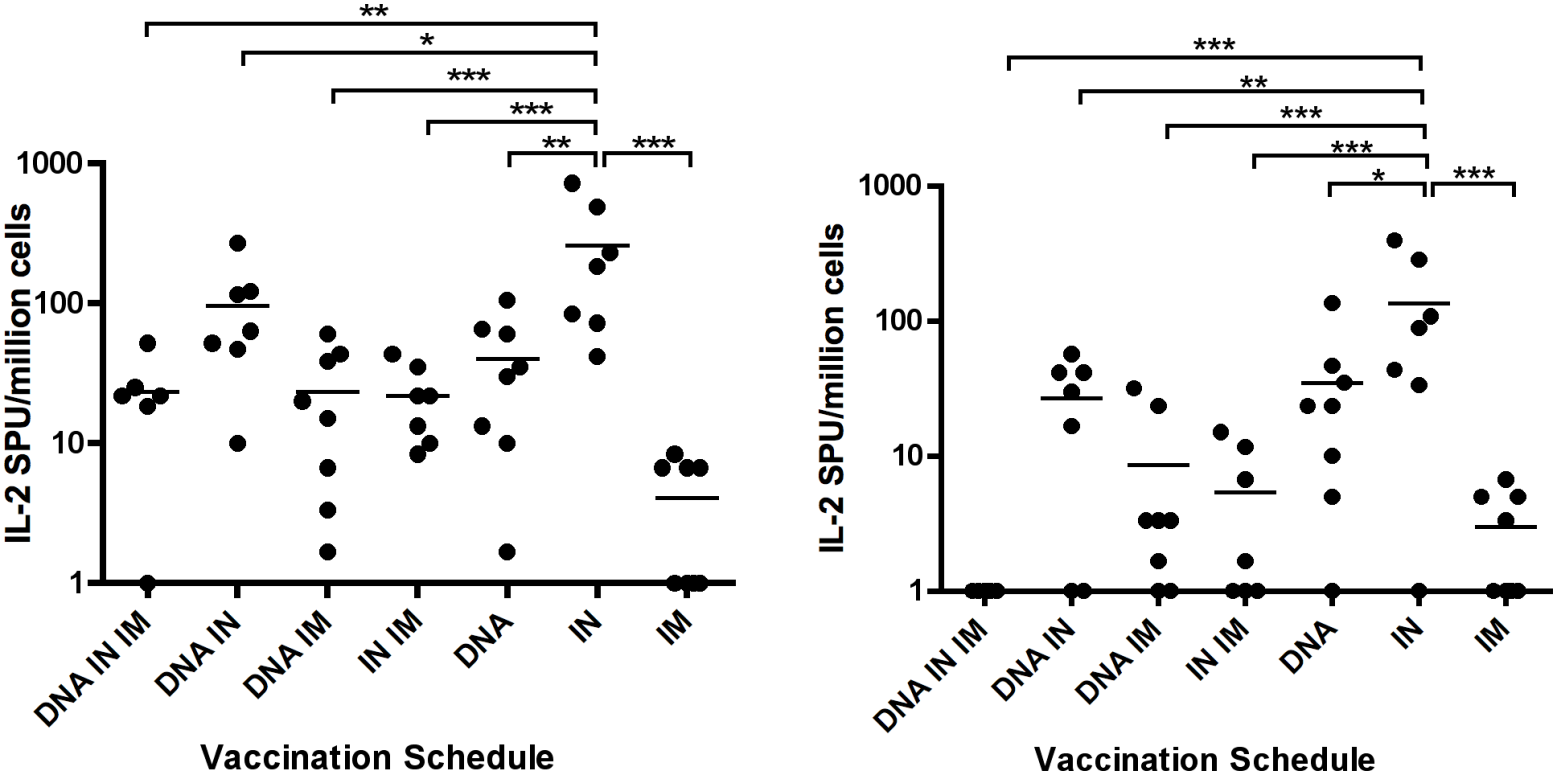
IN vaccination induces potent IL-2 responses in splenocytes after stimulation with HIV-1 Env peptides. Animals were vaccinated three times by the routes indicated. One week after the final vaccination splenocytes were cultured with two HIV Env-specific peptide pools, and IL-2-producing cells were measured using ELIspot kits. Negative responses were assigned a value of 1 to allowing plotting on a log scale. One-way ANOVA with Tukey’s post test was used to analyse data * P <0.05, ** P < 0.01, *** P < 0.001.

As described for IFNγ ELISpots, splenocytes were cultured with HIV-1 Env specific peptide pools and IL-2 producing cells were detected. Again responses to pool 1 were higher in all groups compared to pool 2. The highest frequency of IL-2 positive cells was observed in the IN only vaccination group with mean 259.5 SFU ± 95.54 (pool 1) and 136.2 SFU ± 55.62 (pool 2) observed. This group had significantly more IL-2+ cells than all other groups, showing that combining the IN route with DNA or IM protein reduced the frequency of IL-2 responding cells after three vaccinations. Lowest frequency responses were observed in the IM protein vaccination group 3.542 SFU ± 1.352 (pool 1) and 2.5 SFU ± 0.996 (pool 2), in addition, no response was detected for the DNA + IN + IM group after background was subtracted for pool 2 (Fig 6). This demonstrates that IN immunisation is optimal for inducing IL-2+ cells. Cellular responses are summarised in Table 3.

**Table 3:** Mean cellular responses for each vaccination protocol.

Group	B-cell ELIspot		IFNγ ELIspot		IL-2 ELIspot	
	(SFU/10^6^ cells)		(SFU/10^6^ cells)		(SFU/10^6^ cells)	
	IgG	IgA	Pool 1	Pool 2	Pool 1	Pool 2
DNA IN IM	117.5	65.8	217.3	35.0	23.1	0.0
DNA IN	138.7	**174.1**	113.3	85.2	96.7	26.7
DNA IM	123.5	13.0	290.4	48.1	23.5	8.3
IN IM	96.6	31.2	12.7	9.2	21.9	5.0
DNA	17.5	22.7	**575.0**	**193.1**	40.0	34.8
IN	106.3	111.3	190.4	109.0	**259.5**	**136.2**
IM	200.2	31.7	9.6	1.5	3.5	2.5

### DNA and IN vaccination induce distinct cytokine and chemokine profiles

A multiplex bead assay was used to analyse cytokines released upon stimulation of splenocytes with recombinant HIV-1 CN54 gp140. Cells were stimulated with 5μg/ml gp140 protein, and supernatant harvested after 3 days to assess the induced secretion of 23 murine cytokines. Of these IL-1α, IL-1β, IL-3, IL-5, IL-6, IL-9, IL-10, IL-12p40, IL-12p70, G-CSF, GM-CSF, and TNFα were below the limits of detection and three were constitutively expressed in all groups at similar levels (IL-9, Eotaxin, RANTES) (data not shown).

**Fig 7:**
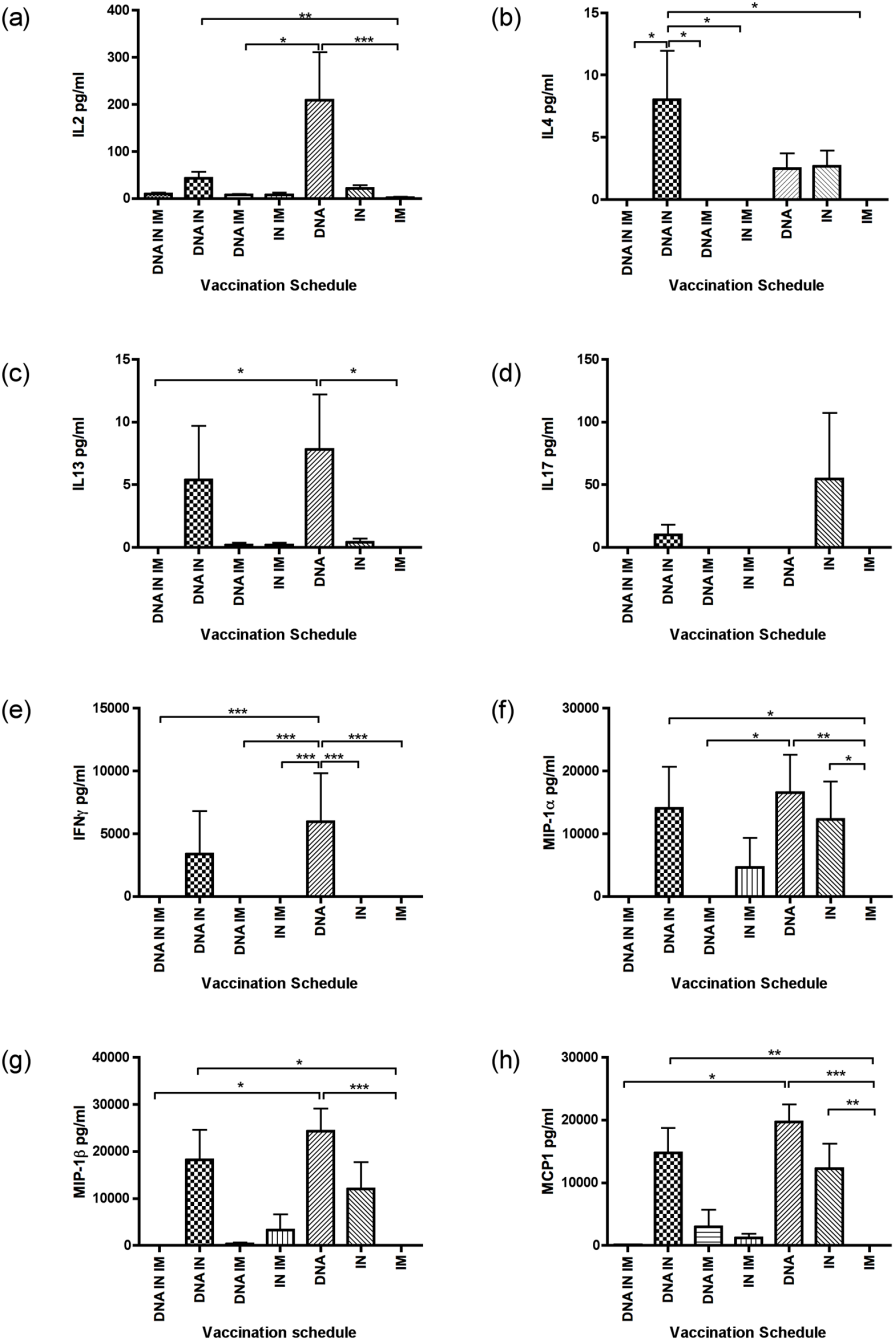
DNA and IN vaccinations induces a distinct cytokine profile compared to IM vaccinations. Cytokines were detected using multiplex bead assay capable of measuring 23 murine cytokines. Differences in cytokine production between groups were observed with selected cytokines IL-2 (a), IL-4 (b), IL-13 (c), IL-17 (d), IFNγ (e), MIP-1α (f), MIP-1β (g) and MCP1 (h). Kruskal-Wallis analysis with Dunn’s multiple comparison test was used to analyse data, * P <0.05, ** P < 0.01, *** P < 0.001.

Significant differences between groups were observed in secretion of IL-2, IL-4, IL-13, IFNγ, MIP-1α MIP-1β and MCP1. Differences were also observed in IL-17 secretion, although this was not significant. The highest levels of IL-2 were observed in DNA, IN and DNA+IN combination vaccination groups with the lowest in the IM protein only vaccination group, in agreement with IL-2 T-cell ELISpot data. IL-2 secretion was significantly higher in DNA alone group, compared to IM protein and DNA + IM protein. IL-2 responses were also significantly lower in the IM protein group compared to DNA+IN immunisation (Fig 7a). ELIspot data indicated that IN vaccination induced the highest frequency of IL-2 producing cells, whereas Luminex data suggested IL-2 secretion was highest after DNA vaccination, this difference may be related to the difference in stimulation used in the two assays: peptide stimulation was used for the ELIspot assays, whereas Luminex data was collected after whole protein stimulation. Induction of IL-4 was observed in DNA + IN, DNA, and IN groups and was significantly higher in the DNA + IN group compared to all other groups except DNA and IN alone (Fig 7b). Induction of IL-13 was also observed in DNA and DNA + IN groups, and was significantly higher in the DNA alone group compared to IM protein, and DNA + IN + IM protein groups (Fig 7c). IL-17 production was detected in DNA and DNA + IN groups, but differences did not reach significance (Fig 7d). In agreement with IFNγ ELISpot data, DNA alone vaccination induced the highest amount of IFNγ, and this was significantly higher than IN or IM protein alone groups, DNA + IM protein, and DNA + IN + IM protein groups (Fig 7e). The beta chemokines MIP-1α and MIP-1β were detected in response to DNA, IN, DNA + IN, and IN + IM protein immunisations. DNA alone and DNA + IN groups had significantly higher levels of both chemokines compared to the IM protein vaccination group. Combination of DNA + IM protein, and DNA + IN + IM protein resulted in very low levels of MIP-1α and MIP-1β (Fig 7f and 7g). Induction of MCP-1 was highest in DNA, IN and DNA + IN groups, and significantly higher than that seen in the IM protein only group. Overall, these data suggest that DNA, IN and DNA + IN vaccinations result in distinct cytokine profiles in comparison to IM protein vaccination, including induction of cytokines (IFNγ, IL-2, IL-4, IL-13) and CCR5 ligands MIP-1α and MIP-1β.

## Discussion

This study investigated whether single modality immunisations or co-immunisation with adjuvanted protein and DNA could efficiently induce cellular and humoral immune responses in systemic and mucosal compartments when administered as part of a conventional prime-boost-boost schedule. Furthermore, we assessed whether the adjuvanted protein administered via a mucosal (IN) or parenteral (IM) route altered the immunological outcome. These data show that IN protein vaccination in the presence of MPLA induces HIV Env-specific humoral and cellular immune responses in the serum and the vagina. Although the use of MPLA as an intranasal adjuvant in humans may face some regulatory hurdles, it is not without precedent with at least one human trial reporting the safe use of MPLA as an intranasal adjuvant for Norwalk virus like particles [22]. In contrast the standard IM protein injection poorly induces IgA or cellular responses, while DNA induces only low-level antibody responses, in agreement with previous data from our group and others [9, 10, 23]. Although not significantly higher than those induced by IN vaccination alone, addition of DNA increased antibody responses 1.5-fold compared to IN alone, reduced the strong Th2 bias of IN induced responses, and resulted in distinct cytokine secretion profiles, that may allow fine tuning of protective responses.

Recent studies in mice, rabbits and macaques have indicated that co-immunisation with HIV or SIV DNA and viral protein delivered intramuscularly or intradermally resulted in increased antigen-specific IgG compared to protein alone, increased neutralisation potency, and preserved cellular responses [18–20]. A further study showed that co-immunisation with DNA and inactivated virus particles effectively induced protection from intrarectal heterologous virus challenge and control of viraemia in a macaque model [24]. These studies support the idea that combination of DNA and protein vaccinations can induce desirable vaccine responses. In contrast to these findings, we saw similar levels of IgG in the DNA + IM group to those observed with IM vaccination alone. This may result from differences in the adjuvant formulation between the studies or may suggest that the adjuvanted IM protein vaccination given here induced maximal responses, so no additional effect was noted with DNA. One significant confounder maybe differences in dose/volume per body mass between mice and larger animals. The roles of IN vaccination and IgA responses were not examined in these previous studies, here we show that both IN and DNA + IN immunisation induce IgA in the serum and vagina, with an increase in IgA in both compartments when DNA is combined with IN immunisation. Data from *in vitro* studies show that HIV-1 Env-specific mucosal IgA can block HIV entry and transcytosis, [25–28] and even low level nAb titres can be sufficient to reduce infection by repeated low-dose challenge [29], thus induction of antigen-specific IgA in the vagina may be a desirable outcome for an HIV vaccine.

Induction of vaginal IgG and IgA responses after IN immunisation supports the idea of the Common Mucosal Immune System composed of the respiratory and genital tracts, at least in mice [9, 10, 23]. Further work will be required to determine the extent to which observed vaginal humoral responses following IN vaccination reflect serum transudation of elicited responses and/or homing of antigen specific B cells to vaginal mucosa.

In agreement with Jalah *et al* [18] we did not observe major differences in antibody avidity between the co-immunisation protocols (S4 Fig), although this was noted by another group [20]. Analysis of DNA prime-protein boost regimes by our group have shown that inclusion of DNA in such regimes induces higher avidity antibody responses [11] particularly by the subcutaneous (SC) route. As only IN and IM routes were used here, it cannot be ascertained whether the simultaneous delivery of DNA and SC protein would similarly increase avidity.

IgG subclass analysis revealed that vaccine modality influenced Th1/Th2 bias of induced responses. The development of Th1 responses, as observed with DNA vaccination, is critical for recovery from infection by influenza [30, 31], RSV [32] and Leishmania [33], although whether this is required for protection/clearance of HIV remains to be seen. Whilst IN vaccination induced strong Th2 biased responses, addition of DNA to this regimen did reduce this bias, but did not switch the profile to a Th1-type response. BALB/c mice are known to have a strong Th2 bias compared to other mouse strains, [34], but the trend for these vaccines to induce Th1 or Th2 polarised responses is likely to be similar in other mouse species. In addition to IgG subclass analysis, Th1 bias of DNA vaccination was also indicated by strong IFNγ responses. DNA vaccinations utilise cellular pathways to process and present plasmid-encoded genes to CD8 T-cells, as transfected cells will transcribe and translate vaccine-encoded proteins. Such proteins are then processed and presented via the MHC class I pathway, eliciting potent CD8+ CTL responses, which are known to be important in controlling HIV replication [35–37]. Secretion of vaccine-encoded proteins from the cell can lead to MHC class II presentation, and induction of CD4 T cell responses [38].

Some previous studies have noted that co-immunisation results in immune suppression, resulting in impaired cellular responses [39, 40]. Such suppression resulted from the induction of CD4+ CD25- FoxP3+ Tregs, which expressed elevated levels of IL-10, and reduced IFNγ and IL-2. These responses resulted in T cell anergy and control of allergen-induced immediate hypersensitivity [41] and required highly antigenic epitopes for induction of Tregs [42]. In this study, cytokine responses after antigen stimulation revealed significant differences in secretion of a number of cytokines and chemokines (see below), indicating the route and modality of vaccination influenced T-cell responses. However, we did not observe significant impairment of cellular IFNγ responses when protein only (IN or IM) were combined with DNA (IN + DNA, IM + DNA), nor were IL-10 responses detected in co-immunisation groups. These data argue against an active suppression of antigen specific cellular responses, rather a modulation of their cytokine profile. Further analysis of the T cell phenotype induced by these vaccination regimes would clarify the mechanism underlying changes in cytokine secretion profiles.

Luminex analysis of antigen-stimulated splenocytes identified distinct cytokine and chemokine secretion between the vaccination strategies. Both DNA alone and DNA + IN vaccination induced IL-4 and IL-13, cytokines known to promote B-cell activation, growth, differentiation and class switching. Release of IL-17 in IN and DNA + IN protocols was also noted, as seen previously [9]. Th17 cells are a unique mucosal CD4 effector population responsible for protection from extracellular bacteria, fungi and viral infection at mucosal surfaces, and these cells are rapidly depleted in HIV infection [43]. They are known to induce antigen-specific B-cell proliferation *in vitro*, and trigger antibody production [44]. Generation of Th17 CD4 T cells is also essential for the formation of follicular helper CD4 T cells, (T_FH_ CD4 T cells) which can promote IgA class switching [45], can be a source of IFNγ, IL-4, IL-13 and IL-17, and are found within the spleen [46]. The presence of IL-17 secreting cells in the DNA + IN and IN vaccinated animals may indicate a mechanism that accounts for the higher levels of IgA induction in the serum and vagina of animals in these groups.

Induction of the CCR5 ligands MIP-1α (CCL3) and MIP-1β (CCL4) were noted in DNA, DNA + IN, IN + IM and IN groups. These cytokines are the natural ligands of CCR5, a co-receptor for HIV target cells entry, and act as a chemoattractant for monocytes, T cells and eosinophils. Expression of MIP-1α and MIP-1β can inhibit infection by R5 strains of HIV [47] and can restrict virus dissemination at early stages of HIV disease [48]. Polyfunctional CD8 T cells expressing IFNγ, IL-2 and MIP-1β correlate with lower viral load in non-progressors [49]. Although the source of these cytokines was not established here, taken together, these data suggest that the different vaccination modalities, particularly the inclusion of DNA vaccination lead to expression of cytokines that promote B-cell function, generation of T_FH_ cells and induce ligands of CCR5 that can reduce HIV infection.

In this study we have compared single vaccination modalities and routes with co-immunisations to determine an optimal regime to induce cellular and humoral responses. We cannot exclude that use of the same products used sequentially and/or in different combinations may have provided additional optimization of response. However the focus of our study was to assess the potential benefit and/or redundancy of combined immunizations given in a conventional prime-boost-boost schedule. Previous studies have shown that DNA can successfully be combined with IM protein vaccination to induce cellular and humoral responses, here we show that DNA co-immunisation can also be combined with mucosal vaccination by the IN route, and that this regimen can increase antigen specific IgG and IgA in the serum and at distal mucosal sites, preserve cellular responses, promote a balanced Th1/Th2 response and induce chemokines and cytokines that can shape antigen specific responses. These data support the idea that DNA and protein co-immunisation by a mucosal route could have desirable effects on vaccination outcome, and is a promising strategy for delivering candidate HIV vaccines.

## Supporting Information

S1 Dataset Dataset contains the raw data values for each Figure listed above, each tab contains the data for one Figure. (XLSX)

S1 Fig Induction of serum IgG and IgA at week 1 and week 4. Mice were vaccinated three times with the indicated vaccine combinations, serum samples were taken 1 week after each vaccination and tested for antigen-specific IgG at week 1(a) and week 4 (b) and IgA at week 4 (c) by ELISA.
(TIF)

S2 Fig IgG subclass analysis after first and second vaccinations. Levels of IgG1 and IgG2a were assessed in serum one week after vaccination 1 (a, b), and vaccination 2 (c, d) by ELISA. IgG1:IgG2a ratios were calculated for each time point (b, d).
(TIF)

S3 Fig Positive correlations were noted between serum antibody titer and frequency of antigen-specific antibody producing cells measured by ELIspot. Two-tailed Pearson correlation analysis was performed on data from all groups.
(TIF)

S4 Fig Similar antibody avidity was observed across all vaccination protocols. The Avidity of antigen specific IgG generated in each group was measured by a urea based ELISA assay and the avidity index calculated.
(TIF)
