# Supplementary figures and images for: Plasmid DNA Vaccine Co-Immunisation Modulates Cellular and Humoral Immune Responses Induced by Intranasal Inoculation in Mice
Source: PLoS One. 2015 Nov 6;10(11):e0141557. doi: 10.1371/journal.pone.0141557 (PMC4636430; doi:10.1371/journal.pone.0141557)

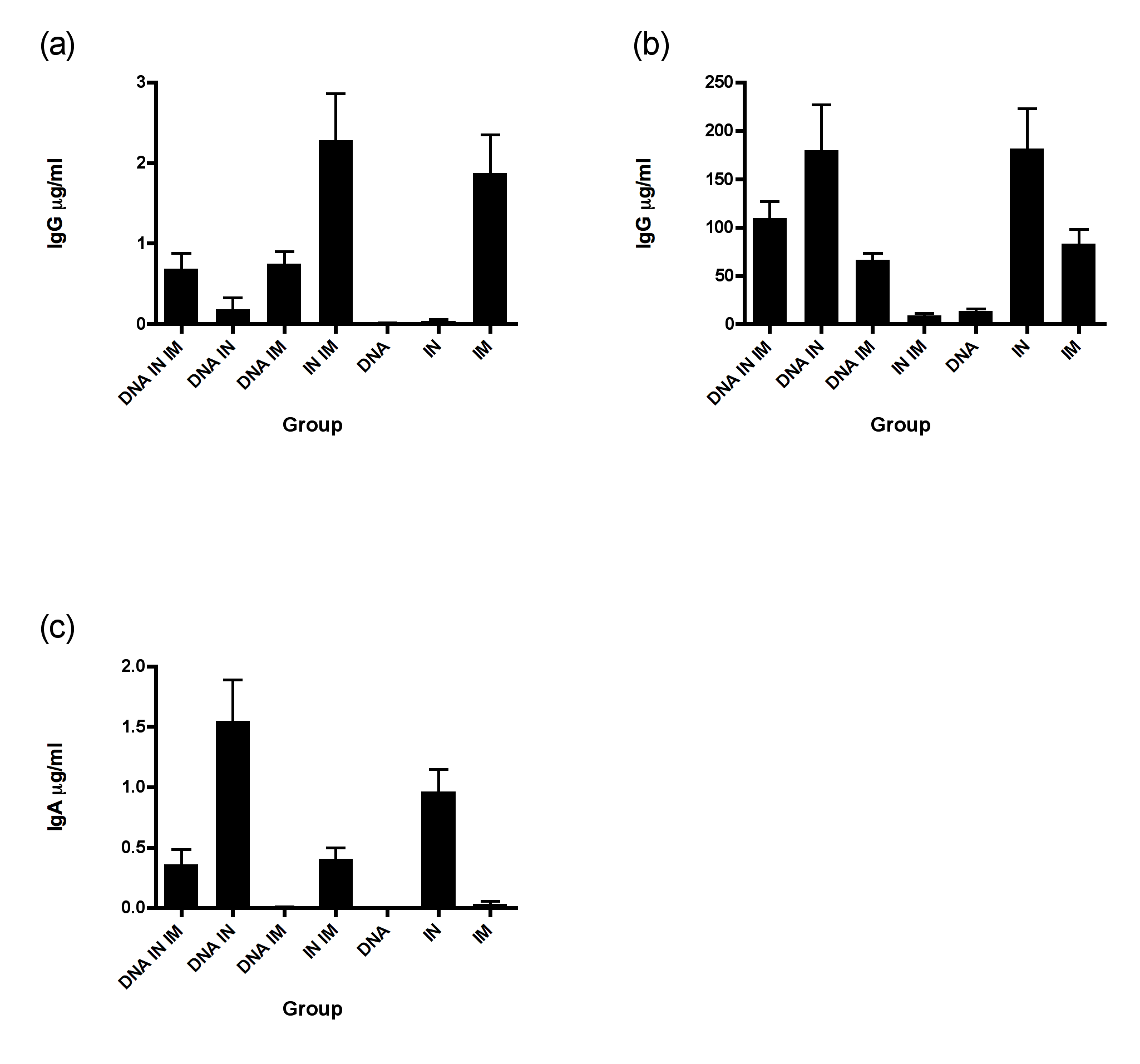

Supplement: S1 Fig — Mice were vaccinated three times with the indicated vaccine combinations, serum samples were taken 1 week after each vaccination and tested for antigen-specific IgG at week 1(a) and week 4 (b) and IgA at week 4 (c) by ELISA. (TIF) [file pone.0141557.s002.tif]

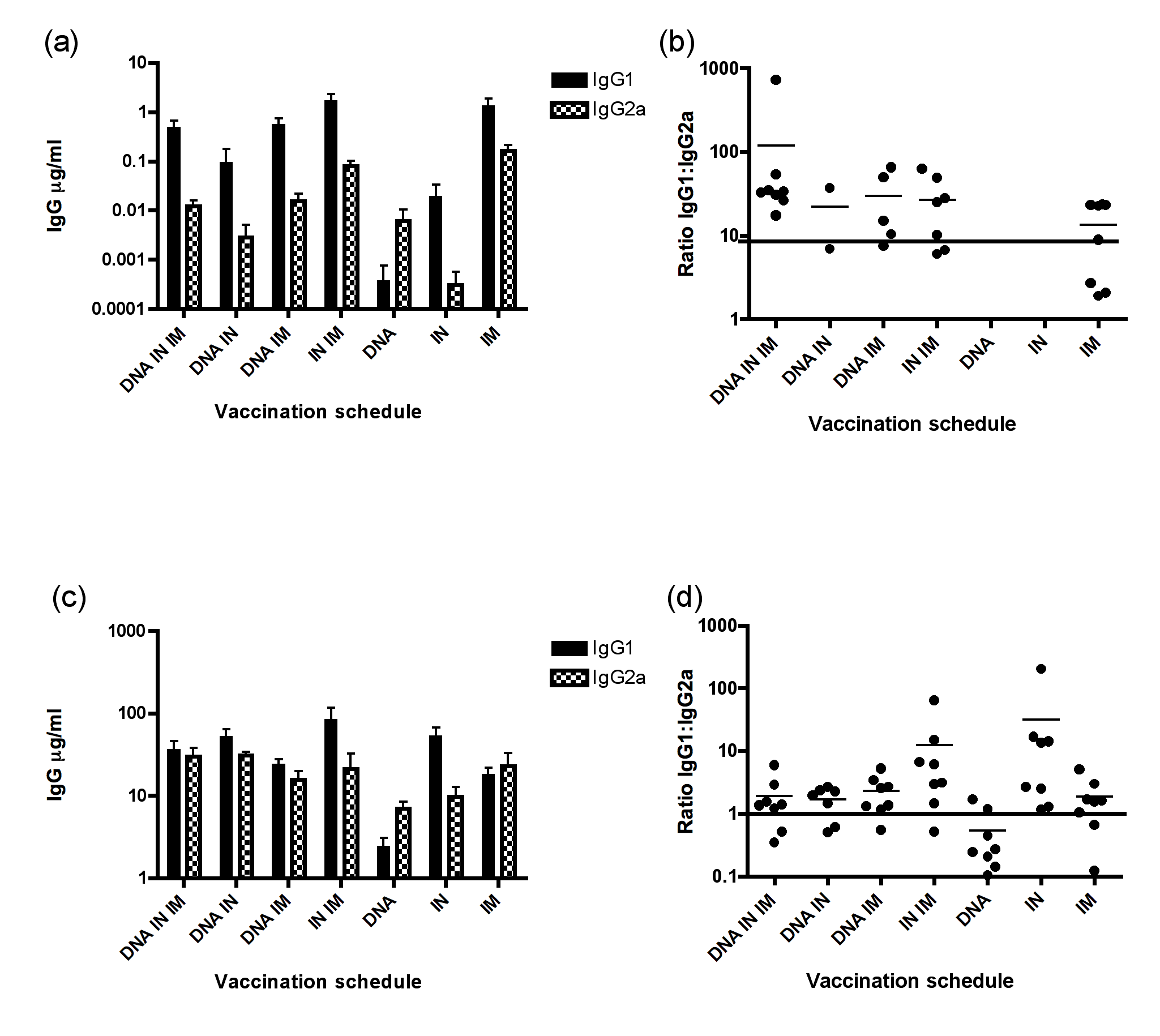

Supplement: S2 Fig — Levels of IgG1 and IgG2a were assessed in serum one week after vaccination 1 (a, b), and vaccination 2 (c, d) by ELISA. IgG1:IgG2a ratios were calculated for each time point (b, d). (TIF) [file pone.0141557.s003.tif]

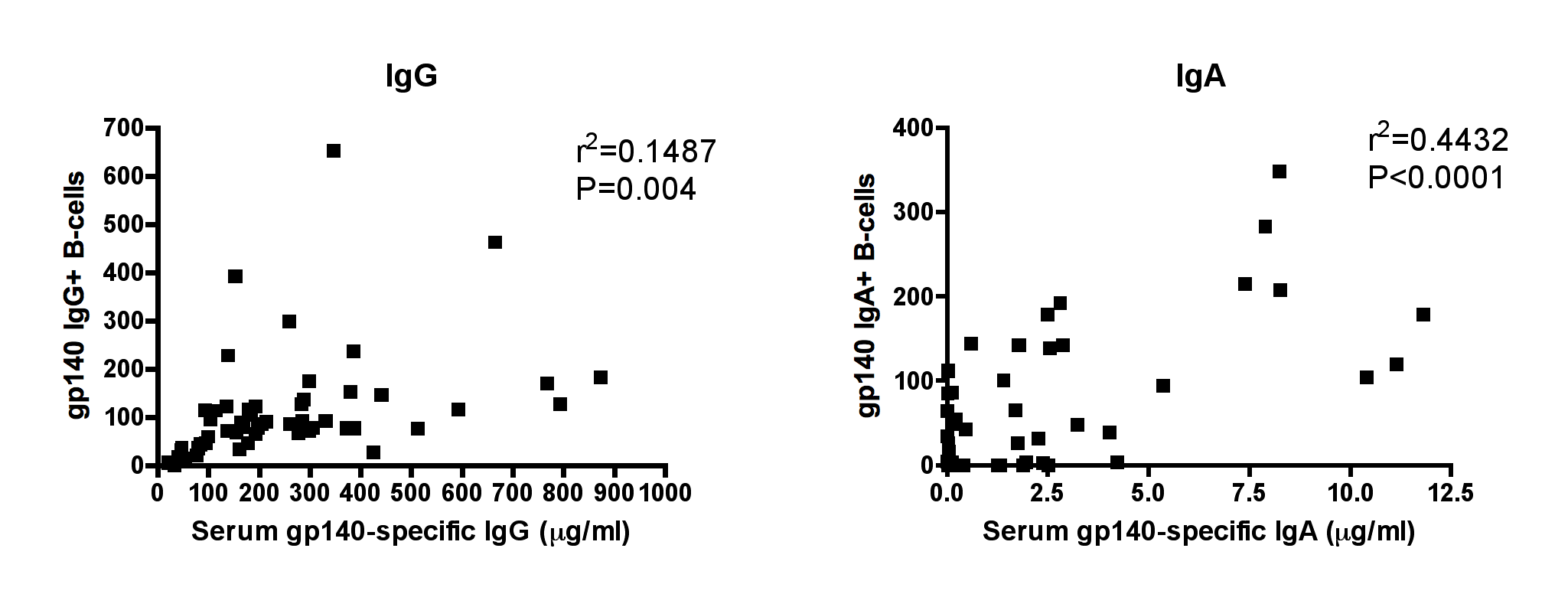

Supplement: S3 Fig — Two-tailed Pearson correlation analysis was performed on data from all groups. (TIF) [file pone.0141557.s004.tif]

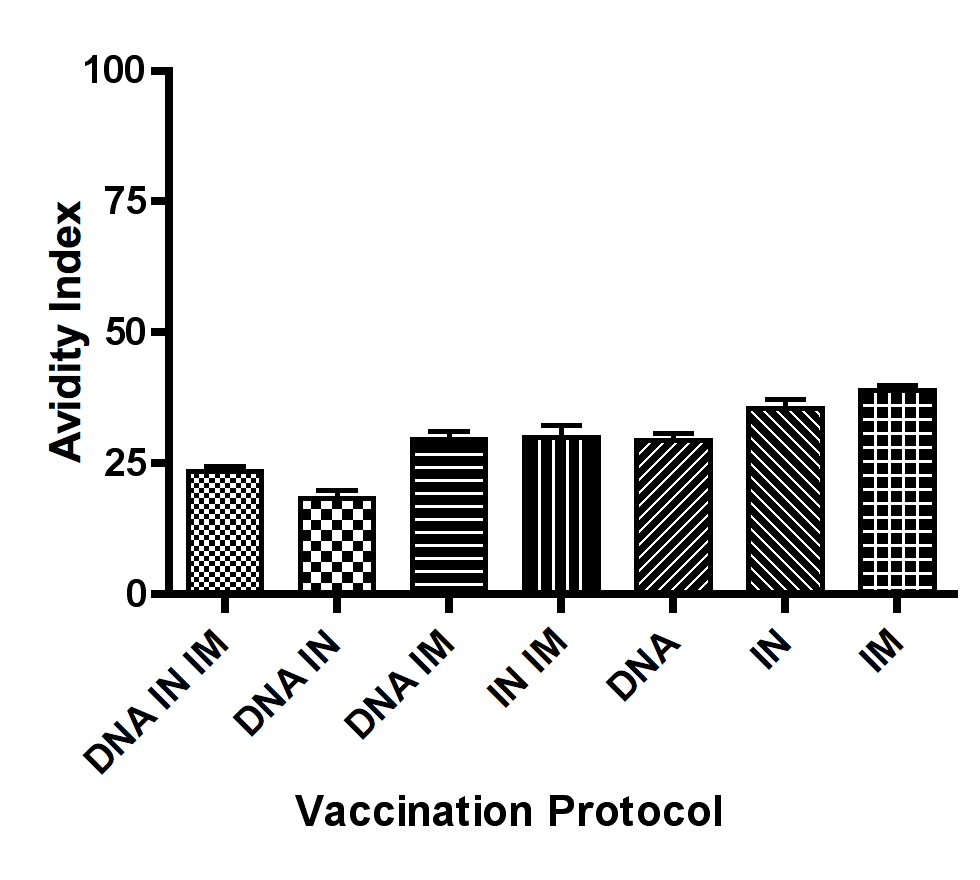

Supplement: S4 Fig — The Avidity of antigen specific IgG generated in each group was measured by a urea based ELISA assay and the avidity index calculated. (TIF) [file pone.0141557.s005.tif]
